# Primary Uterine Cervix Schwannoma: A Case Report and Review of the Literature

**DOI:** 10.1155/2012/353049

**Published:** 2012-12-20

**Authors:** Maryam Tahmasbi, Johnny Nguyen, Masoumeh Ghayouri, Yuan Shan, Ardeshir Hakam

**Affiliations:** ^1^Department of Anatomic Pathology, H. Lee Moffitt Cancer Center and Research Institute, Tampa, FL, USA; ^2^Department of Pathology and Cell Biology, University of South Florida, Tampa, FL 33612, USA

## Abstract

Schwannoma (neurilemmoma) is a benign peripheral nerve sheath tumor that occurs in a wide variety of locations; however, its finding in the uterine cervix is extremely rare. We report a case of an incidental primary benign cervical schwannoma in a 48-year-old woman. In the English literature, a few cases of primary schwannoma of the cervix have been reported, which include seven cases of primary malignant cervical schwannoma and only two that are benign. These cases are reviewed in the following discussion.

## 1. Introduction

Cervical schwannoma is a very rare entity in the field of gynecologic pathology. In this paper, we describe an incidental benign cervical schwannoma in a 48-year-old woman. 

## 2. Case Presentation

A 48-year-old woman presented to the emergency department with a sharp, severe left lower quadrant pain. Ultrasound imaging of the abdomen and pelvis demonstrated an enlarged uterus with multiple masses (highly suggestive of leiomyoma), an 8.7 cm left adnexal complex mass, and minimal fluid in the pelvis. Subsequent computed tomography (CT) scan of the abdomen and pelvis confirmed the above findings.

Pelvic examination revealed a posterior cervix with an enlarged, boggy uterus, but no tenderness. The cervix could not be visualized on speculum exam due to a distorted uterus. No palpable inguinal lymph nodes were identified. The remainder of the physical exam was unremarkable. Laboratory workup was notable for an elevated CA-125 level (125.9 U/mL).

The patient's past medical history was significant for breast cancer status after lumpectomy, radiation therapy, and Tamoxifen therapy. Her past gynecologic history was remarkable for laparoscopic myomectomy (12 years prior to this encounter). She resumed having regular cycles every 30 days and denied any recent abnormal bleeding, weight loss, and gastrointestinal or genitourinary symptoms.

The patient was advised that the best approach would be to remove the uterus and ovaries and send them for intraoperative consultation during the surgery. Subsequently, the patient underwent a total abdominal hysterectomy and bilateral salpingo-oophorectomy (TAH/BSO).

Exploration of the pelvis at the time of surgery revealed a large multilobated uterus that was about 16 weeks in size, as well as hemorrhagic cysts on both ovaries. The left ovary was no longer 8 to 9 cm, but was instead around 4 to 5 cm. Additionally, there was also some blood in the pelvis. Pelvic washings were obtained, and a representative section from the left ovary was submitted for frozen section examination. The diagnosis was reported as a benign hemorrhagic cyst. The surgery and the postoperative course proceeded without complication and the patient was discharged home on postoperative day 4.

## 3. Pathologic Findings

On gross examination the left ovary was remarkable for a 2.0 × 1.8 × 1.4 cm hemorrhagic cyst containing necrotic material. The right ovary was remarkable for a 2.5 × 2.0 × 1.5 cm hemorrhagic cyst. Both fallopian tubes were unremarkable.

The enlarged and distorted uterus (628.9 gram and 17.8 × 11.0 × 10.5 cm) had multiple subserosal, intramural, and submucosal leiomyomas ranging from 0.1 to 4.0 cm in greatest dimension. Also, within the uterine fundus attached to the anterior and posterior walls were multiple tan-pink focally hemorrhagic fleshy polyps ranging from 5.0 to 2.7 cm in greatest dimension, which all appeared to be confined to the mucosa.

Gross examination of the cervix revealed a 0.9 × 0.4 × 0.3 cm rubbery nodule on the posterior endocervical canal which had a homogenous gray-white cut surface. There was also a 1.3 × 0.4 × 0.4 cm tan-white soft polyp arising from the anterior endocervical canal. The remainder of the cervix was unremarkable.

**Figure 1:**
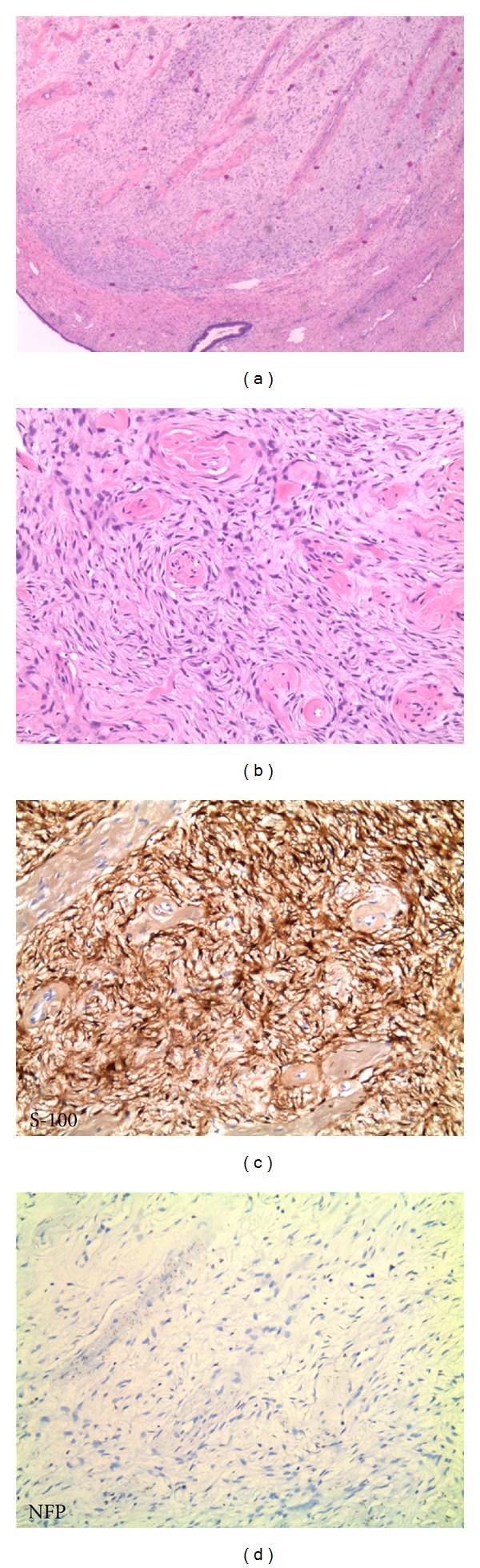
(a) Low-power view (2.5x) of the lesion showing a well-circumscribed mass with spindle cell proliferation and thick-wall hyalinized blood vessels. (b) Higher power view (10x) of the lesion. (c) Spindle cell component with strong S-100 immunoreactivity (10x). (d) Negative stain for NFP (10x).

Histologically, the white rubbery nodule of the endocervical canal was a well circumscribed lesion composed of a spindle cell proliferation with localized nuclear palisading and abundant thick-wall hyalinized blood vessels (Figures 1(a) and 1(b)). Mitotic activity was minimal, which was confirmed with a scant Ki-67 staining pattern. Immunohistochemical stains with adequate controls showed strong immunoreactivity for S-100 (Figure 1(c)). However, the tumor cells were negative for NFP (neurofilament protein) (Figure 1(d)), CD34, desmin, actin, chromogranin, synaptophysin, and pan-melanoma. The combination of morphological and immunohistochemical features suggest a neoplasm of neural origin, specifically, schwannoma.

Additional microscopic findings were ovarian endometriosis, hemorrhagic corpus luteum, uterine leiomyomas and adenomyosis, endometrial polyps, and a benign endocervical polyp.

## 4. Discussion

Schwannoma (neurilemmoma) is a benign peripheral nerve sheath tumor that occurs in a wide variety of locations. Most commonly they occur in middle age, and without a clear sex predilection. They usually present in superficial soft tissues of the extremities, perhaps relatively most often in the head and neck region and the distal parts of the extremities. Certain schwannomas, especially the bilateral vestibular ones, are associated with neurofibromatosis type 2 (NF2) syndrome and hereditary NF2 gene mutations. Uncommonly, large tumors are found in the posterior mediastinum or the retroperitoneum. Typical schwannomas can also involve visceral sites primarily, such as the GI tract, kidney, and breast; however, its finding in the uterine cervix is extremely rare. 

In the English literature there have been less than ten reports of a primary schwannoma of the uterine cervix listed in Table 1 (most of which were diagnosed as malignant schwannoma), one pigmented melanocytic schwannomas, and only two benign cervical schwannomas. To the best of our knowledge, this case represents the third reported incident of a benign schwannoma of the cervix.

Gwavava and Traub reported the first case in 1980 [1]. The patient was a 38-year-old woman who was referred to a gynecology clinic on account of a firm nodular swelling on the anterior lip of cervix. No other pelvic abnormality was detected. The tumor was removed by diathermy excision and the morphology was that of a classic schwannoma with Antony A, Antony B and Verocay bodies. No follow-up information on this patient was given.

The clinical presentation in the second reported case of benign schwannoma by LeMaire et al. in 2002 [2] was also very similar to our case. The case was an asymptomatic 47-year-old woman who was found to have a tumor on the posterior lip of the cervix on a routine annual pelvic exam. The tumor was excised using loop electrosurgical excision procedure (LEEP) and found to be a benign schwannoma. Subsequent CT scan of the pelvis failed to reveal any other abnormalities and no further treatment was contemplated. 

The first case of pigmented melanocytic schwannoma of the uterine cervix was reported in 1990 by Terzakis et al. [3]. It was a 47-year-old woman with a lesion of the cervix that presented clinically as a protruding or aborted leiomyoma. Histologically, the tumor was a spindle-cell neoplasm with localized nuclear palisading composed of cells with abundant pink cytoplasm that contained considerable brown melanin granules confirmed by Fontana's stain. Cytologically nuclear pleomorphism, hyperchromatism, giant nuclear forms, and mitoses were observed and tumor cells showed a positive reaction to S-100 protein and HMB-45. After 3 years follow-up the patient was disease free with no evidence of metastasis.

The largest series of the malignant schwannoma or malignant peripheral nerve sheath tumor (MPNST) was reported by Keel et al. in 1998 [4]. In this study, the patients were 25, 65, and 73 years old and two of them presented with abnormal vaginal bleeding. Two tumors were polypoid and one was ulcerated and they measured 1.3, 4.4, and 5.0 cm in dimension. Histologically, the tumors were composed of hypercellular and hypocellular zones of fascicular spindle cells, similar to their soft tissue counterparts. All tumors were mitotically active and were positive for S-100 protein and negative for muscle markers. Follow-up in two patients showed no evidence of disease at 15 months and multiple abdominal metastases 2 years post-hysterectomy, respectively.

Prior and after the Keel's report, five more cases of the malignant schwannoma of the cervix were reported (the first one in 1988 and the last one in 2005) that all of them were presented with the similar clinical presentation of abnormal vaginal bleeding. The patients were 47, 41, 51, 65, and 27 years old. The tumors were ranging from 1 to 4.4 cm in dimension, and histologically all of them were composed of mitotically active hypercellular schwannoma with nuclear atypia [5–9]. 

Lesions that should be considered in the differential diagnosis in this case are neurofibroma, leiomyoma, angiomyofibroblastoma, and desmoplastic melanoma. The absence of staining for CD34 and NFP, but strong positivity for S-100, supports the diagnosis of schwannoma as opposed to neurofibroma. Moreover, the presence of thick-wall hyalinized blood vessels favors the diagnosis of schwannoma. Positivity for S-100 protein and negativity for desmin and actin argue against leiomyoma and angiomyofibroblastoma. Finally, a negative pan-melanoma stain essentially excludes desmoplastic melanoma.

## Figures and Tables

**Table 1:** Summary of the previously reported primary schwannoma of the uterine cervix.

Case	Clinical presentation	Age	Pathological findings
Gwavava and Traub (1980) [1]	Firm nodular swelling on the anterior lip of cervix	38	Classic schwannoma with Antony A, Antony B, and Verocay bodies
LeMaire et al. (2002) [2]	Asymptomatic, incidental finding on a routine annual pelvic exam	47	Benign schwannoma
Terzakis et al. (1990) [3]	Presented as a protruding or aborted leiomyoma	47	Pigmented melanocytic schwannoma
Keel et al. (1998) [4]	Two of three reported cases presented with abnormal vaginal bleeding, two of them were polypoid lesions, and one was ulcerated	25 65 73	Malignant schwannoma, malignant peripheral nerve sheath tumor (MPNST)
Sloan (1988) [5]	Abnormal vaginal bleeding	47	Malignant schwannoma
Junge et al. (1989) [6]	Abnormal vaginal bleeding	41	Malignant schwannoma
Lallas et al. (1999) [7]	Abnormal vaginal bleeding	51	Malignant schwannoma
Bernstein et al. (1999) [8]	Abnormal vaginal bleeding	65	Malignant schwannoma
Giovannantonio et al. (2005) [9]	Abnormal vaginal bleeding	27	Malignant schwannoma
